# Different Shades of Beauty: Adolescents’ Perspectives on Drawing From Observation

**DOI:** 10.3389/fpsyg.2020.00687

**Published:** 2020-04-21

**Authors:** Nurit Wolk, Adi Barak, Dani Yaniv

**Affiliations:** ^1^Ono Academic College, School of Society and the Arts, Kiryat Ono, Israel; ^2^The Louis & Gabi Weisfeld School of Social Work, Bar-Ilan University, Ramat-Gan, Israel; ^3^Emili Sagol Creative Arts Therapies Research Center, The Graduate School of Creative Arts Therapies, University of Haifa, Haifa, Israel

**Keywords:** art therapy, drawing, mindfulness, observation, adolescence

## Abstract

**Background:**

Drawing from observation (DFO) is an art therapy method that entails drawing an object, along with guided reflections on process and outcome. In this qualitative study, we explored adolescents’ perspectives on their DFO experience, and how they perceive it as having influenced their emotional well-being.

**Methods:**

We interviewed 10 adolescents who participated in a DFO group, regarding their perspectives on DFO. Participants were asked to refer to their experience, as well as to provide examples of their drawings. Through a thematic analysis we integrated data from interviews and drawings.

**Results:**

Adolescents experienced three lines of tension in DFO: Between self-acceptance and self-judgment, between merging and separation, and between similarities and differences. Processing these tensions has the potential to increase their emotional well-being.

**Conclusion:**

DFO could make a meaningful contribution to adolescents’ emotional well-being. The unique intersection between object, observation, and drawing in art-therapy addresses adolescents’ emotional-developmental challenges.

## Introduction

### Drawing From Observation as Therapy

The art therapy literature provides various definitions for the concept of drawing from observation (DFO; Monahan, 2016). With regard to therapy, DFO can be broadly defined as a creative arts therapy (CAT) method that is based on the drawing of an external object, such as a model, a still life, or any other visual object, along with guided reflections on the process and/or product of drawing (see, Hoptman, 2002; Newman, 2003; Ashwin, 2016).

When DFO is facilitated in a therapeutic setting, participants can gain insight into a variety of personal issues. As DFO is never merely copying, participants ultimately explore how the external object, and the drawing itself, might be a projection of their inner self and/or inner consciousness (Ramm, 2005). Thus, according to Friedl Dicker-Brandeis, the participants in a DFO session “are invited to seek the subject’s essence, to see it both inside and out, to become one with their subject, to empathize with it” (Elsby, 2012, p. 1). The product and process of drawing, in this sense, become the means for self-exploration.

Wix (2009), in this regard, offered the term “empathic aesthetic.” According to Wix, DFO promotes the observer’s ability to project and to have empathy with the other, as well as with the natural world. This process allows for mindful, “transitional” play, creativity and artistry, both during the creative process *and* when looking at the end product (Milner, 2005a, b). Cognitively speaking, an “empathic aesthetic” calls for drawing without exclusively relying on top-down processes, such as memories and previous knowledge, and instead emphasizing bottom-up processes, such as current sensations and spontaneity (Yaniv, 2018). Being in such a spontaneous “zone,” participants of DFO sessions may reexamine and process issues related to their emotional well-being.

### Drawing From Observation and Adolescents

Adolescence is a critical age for achieving one’s human potential (Patton et al., 2016). It is therefore crucial to understand the challenges to adolescents’ emotional well-being, a multilayered and complex structure that is affected by various factors. For one thing, familial support and relationships play a primary role in adolescents’ well-being. In this regard, the amount of quality time spent together with family (Offer, 2013), as well as family history – and within it, the existence or absence of adverse childhood experiences (Balistreri and Alvira-Hammond, 2016) – have a crucial influence on future emotional well-being. These issues should be understood against the backdrop of an inherent tension that adolescents experience between striving to separate from their parents and at the same time needing close and supportive relationships with them (Meltzer and Harris, 2011). Thus, being able to negotiate these tensions with parents might result in greater well-being. Social connectedness is also paramount for adolescents’ emotional well-being (Jose et al., 2012; Olsson et al., 2013); that is, the more that adolescents are positively connected to peers, the more they will experience greater emotional well-being, and vice versa. In some cases, however, when social connectedness is achieved through increased social media engagement, this connectedness might lead to feelings of isolation and depression (Best et al., 2014). Finally, future orientation – that is, having a positive vision of a future “possible” self – can influence the present well-being of adolescents (Johnson et al., 2014). These factors are influenced by adolescents’ sense of identity and the amount of acceptance they feel that their emerging identity might receive from others (Barak and Barak, 2019).

Can DFO assist adolescents in negotiating these challenges to their emotional well-being? Only a few accounts describing the significance of DFO for adolescents exist, mainly in the educational field (Drake et al., 2016; Duncum, 2018). These accounts demonstrate how therapists who use DFO during their sessions consider adolescents to be a target group that stands to gain significantly from its use (e.g., Albert, 2010). The combination of educational and therapeutic components in the DFO method is regarded as essential for enabling adolescents to gain different points of view and a broader perspective on their lives (Burton, 1984). Moreover, through these educational processes of DFO, adolescents are invited to be creative without feeling threatened by the therapist’s interpretation or their skill level (Riley, 1999). Shapira (1993), referring to adolescents in a psychiatric ward, claimed that by having them observe a visual image and copying it, they would be encouraged to create a “transitional image” through which they could process their emotions. Through such mechanisms of sublimation, Graham (1994) explained, DFO would allow adolescents a safe way to process traumatic experiences.

The abovementioned studies, while supporting the use of DFO with adolescents who experience mental health challenges (Burton, 1984; Riley, 2001), do not provide us with adolescents’ perspectives on *how* DFO contributes to their emotional well-being. To address this lacuna in the research, we set out to study the unique intersection of object, observation, and drawing in DFO based CAT, from adolescents’ perspectives. Specifically, we asked the following research question: What are adolescents’ perspectives, experiences, and insights regarding participation in DFO sessions? This question was divided into three sub-questions: What are adolescents’ perspectives and experiences regarding (1) the process of observation (2) the process of drawing, and (3) the DFO product, within a therapeutic group.

## Methodology

We used a descriptive qualitative approach (Eisner, 2008), integrating a textual thematic analysis of adolescents’ interviews with examples of drawings. Specifically, Braun and Clarke’s (2006) thematic analysis in psychology seemed most suitable as a flexible method, which could form the basis for textual analysis referring to visual imagery, as is more fully described below.

### Research Process

All participants in this study were Israeli adolescents between the ages of 13–20 who agreed to participate, with a minimum experience of one year and up to three years in DFO groups. These DFO groups were conducted by the first author, between the years 2008 and 2014, in a post-hospitalization therapeutic boarding school and in an alternative, “open” school. Both schools allow their students to choose their own scholastic timetable. During the sessions, the participants in both groups were invited to engage, explore, and express themselves via the practice of DFO.

Drawing from observation sessions lasted for 1 h and were guided by an art therapist (first author). At each session, the art therapist offered guidance for observation, stressing a bottom-up approach. The therapist offered objects to draw at each meeting. She made this suggestion in order to promote listening and self-expression, and to prevent unnecessary pressure; however, participants had the freedom to choose whether to follow the therapist’s guidance or find other ways that suited them (see Table 1).

**TABLE 1:** The outline of a DFO session.

Description of studio space in DFO session	The studio has a table in the center, around which participants work (usually, but not necessarily, seated). At the beginning of each session, the sketchbooks are put out for each participant, together with all the needed materials. The external object of drawing is positioned in a way that allows observation from all parts of the table.
The creative process (35–40 min)	Participants observe and draw; the drawing work is mostly done in sketchbooks. The art therapist’s emphasis is on experiencing and playfulness. However, and especially when members of the group make such requests, the art therapist can include educational elements, such as how to create a shadow, or perspective, and how to integrate elements. Further on, additional educational information regarding the uses of techniques and materials (e.g., coal, pen, ink, etc.) could be offered per request. While participants are drawing, the therapist encourages them to explore different aspects of the process and explore aspects of drawing that are “unknown” to them, thus creating a creative atmosphere. Exploration and curiosity are encouraged. Per the art therapist’s discretion, particular challenges and instructions are given to individual participants as a way of advancing their progress (for instance, participants who are highly critical of their work are encouraged to use scribbling; see Practical Implications section).
Sharing and concluding (10–15 min)	At the end of each session, participants are invited to present their products in an exhibition format (see McNiff, 2004), and encouraged to view their and others’ work and exchange feedback with one another. After the personal interaction concludes, participants are invited to openly discuss the process, the product, and their feelings regarding the whole session with the entire group. During group sharing, the art therapist asks questions about the different ways through which participants chose to express themselves through their drawings. Participants are invited to share their insights regarding their personal discoveries and understandings, as well as their frustrations and questions. The art therapist encourages open interaction between all participants in the group discussion, thus helping the group to reach its full dialogic potential.

All in all, each group held between 30 and 36 meetings, depending on the school’s calendar. Participants were apprised of the timetable and end date at the first group meeting. Each group had one weekly meeting.

Upon receiving approval from the university’s ethics committee, the research team, via the participating organizations, invited adolescents who had participated in CAT DFO sessions at either of the two organizations mentioned previously. Ten participants provided written consent to volunteer and participate in the research. For minors, parental consent was obtained. An interview was then scheduled for each participant; the interviews were conducted by the first author (see, Ethical Considerations section for a discussion of this issue). All interviews were conducted in a single session, lasting between 1.5 and 2.5 h. During the interview, which was semi-structured, participants were asked to refer to their drawings. Most of these drawings were gathered in sketchbooks that had been given to the participants in the period during which they had participated in the DFO sessions. Some drawings and sketchbooks were brought to the interviews by the participants. Other drawings were brought by the interviewer, who kept patients’ drawings per their request, in compliance with the ethical code of the Israeli association of Creative and Expressive Therapies (The Israeli Association of Creative and Expressive Therapies, 2018). Participants were asked to share their drawings and their insights regarding the following topics: their general experience of the DFO sessions; their perspective on the role of observation and the drawing process during DFO; their perspective on specific drawings that had meaning for them (they were also asked to reflect on that meaning, as well as on the process and insights they gained through creating these drawings). All interviews were recorded and transcribed for analysis. Participants gave their written permission for the researchers to use their drawings and data in the analysis and future publications.

### Sample

The sampling strategy in this research study was based on criterion sampling (Patton, 1990). The criterion for inclusion in this research was participation of at least one year in DFO sessions. Nowadays, adolescence is defined as an ongoing developmental period between childhood and adulthood, starting at the age of 10 and ending at the age of 24 (Sawyer et al., 2018). Interviews were held until data collection had reached a point of saturation – that is, the point at which the interviews stopped yielding new insights regarding the identified themes (See, Morse, 1995; see also Bowen, 2008; Hennink et al., 2017). This determination, as Saunders et al. (2018) explained, should be based on the assessment of the researchers, in an ongoing process of examining the data as they accumulate, and concluding that more data will not make a significant contribution to the theoretical understanding already established. Pursuant to this idea, we engaged in such a process until we could determine, based on an ongoing analysis of interviews throughout the data collection phase, that new data would not provide new or meaningful insights into our research questions.

Table 2 summarizes participants’ demographics.

**TABLE 2:** Participants’ demographics (*N* = 10).

Characteristics		%
Gender		
	F	60
	M	40
Age group at interview time		
	under 15	10
	15–18	30
	18–20	60
Age during participation in DFO group		
	13–15	10
	15–18	30
	18–20	60
Group source		
	Democratic school	40
	Therapeutic Boarding School	60
Duration of participation		
	One year	60
	Two years	20
	Four years	20

### Data Analysis

This research was based on a descriptive analysis of participants’ interviews, which included references to the participants’ drawings and allowed them to reflect on their work/process with regards to these drawings. In the presentation of our analysis, we integrated interview data with visual data – to allow for a better understanding of participants’ references to their work.

As noted, analysis of the interviews and drawings was done in accordance with the six steps of thematic analysis as described by Braun and Clarke (2006). Our justification for asking participants about their drawings was based on Gillian’s (2007) third principle of critical visual methodology. The latter states that one must pay maximal attention to participants’ explicit observations and interpretations of their art, while the opinion of the researcher is to be considered as guesswork or speculation (see also Guillemin and Drew, 2010). Based on this idea, interview analyses allowed for an integrated understanding of participants’ experiences with regards to specific drawings. The results of this integrative phase are summarized in the “Findings” section.

The analysis presented in this article is based on the Hebrew transcripts of the interviews. That is, the analysis process was conducted in Hebrew, with only the final research report being translated into English. Thus, translation did not constitute a barrier to understanding. Regarding the presented results, all three authors were responsible for confirming separately that the translation of the results reflected the actual meaning of the original text, and disagreements were discussed until agreement was achieved. The final translation of the quotes was sent to a bilingual language editor who made sure that the language was correct and reflected the actual meaning of the original Hebrew quotes. The revised-edited texts were then sent to the authors for final approval. The results presented in this paper are the end products of this process and reflect the original Hebrew texts as accurately as possible.

Although conducting research on groups facilitated by a particular author could potentially introduce bias into the interpretation process, we made sure that an analysis was conducted by all three authors; disagreements were openly discussed, with the goal of generating a deep, contextualized, and coherent interpretation. In this process of multiple coding, “what is ultimately of value is the content of disagreements and the insights that discussion [between researchers] can provide … alerting researchers to all potentially competing explanations” (Barbour, 2001, p. 1116). Given that DFO is not commonly practiced in Israel, the sample had to be limited to the author’s facilitated groups (see section “Limitations and Conclusions”).

### Ethical Considerations

The university’s ethics committee granted approval to conduct the research. As a DFO research study deals with the therapeutic experience, steps were taken to ensure the well-being of research participants. Participants were approached only after the therapeutic process was completed. An elaborate consent form was presented to the participants, and signing it was obligatory for their participation. Adolescents under the age of 18 were required to provide custodial consent in writing. Participants were invited to participate by a third party (i.e., their educational organization). No direct contact between the art therapist (first author), the other researchers and participants was made before participants’ consent was given.

## Findings

In their interviews, the adolescents in our study described movement across three related dimensions: the aesthetic, mindfulness, and reflection. In all three dimensions, there were evident tensions inherent in the triad of object, drawer, and drawing. Specifically, in the aesthetic dimension participants described a tension between self-acceptance and judgment; in the mindfulness dimension participants experienced a tension between merging (fusion) and separation; and in the reflection dimension participants were challenged with issues of similarities and differences.

### The Aesthetic Dimension: Between Judgment and Self-Acceptance

An aesthetic dimension manifested in participants’ tendency to judge and label the aesthetic quality of their product, as well as by striving to accept the product as it was. This dimension reflected their engagement with self-acceptance, and often began with their judgment of the artistic qualities of their drawing. Self-acceptance, which ultimately followed, was expressed as satisfaction, an acknowledgment of one’s capability despite the inevitable imperfections in one’s work, and a sense of wonder and discovery regarding one’s artistic creation, alongside an understanding that there are many ways to be artistic.

This process can be identified in the words of some of the interviewees, who expressed this tension between their initial tendency to be judgmental of their work and their developing ability to look at their art with joy and excitement. Participant 2 said:

I just like that scribbling with the pencil [doing drawing movements with her hand] and suddenly – it turns out to be beautiful. It’s always such a surprise! Sometimes it’s “Wow” and sometimes it’s less than that, but it’s always prettier than what I thought it would be.

Though sometimes unsatisfied with their drawings, all of the participants described a wish to create a beautiful end-product. This wish often resulted in stress or disappointment. Participant 5, for example, described a sense of frustration that was associated with his striving for perfection:

The number of pages that are thrown away [in a drawing session] is very large. I felt that everything should be perfect. Do you know what? Perfectionism! For me drawing increases the need to be perfect.

Indeed, the aspiration for perfection, which sometimes served as a motivational force, also caused difficulty, frustration, and resentment toward the artistic product. Participant 9’s words illustrate the difficulty in wanting to achieve a perfect aesthetic product:

It is frustrating but in many cases it’s like [a positive] part of the challenge … However, in many cases I think it could cause me to give up on painting.

The judgmental element, accompanied by a sense of worthlessness and frustration, might have led participants to draw in a more casual manner. Scribbling, in this regard, allowed for an expression of worthlessness, but also represented playfulness and freedom – both stemming from relinquishing the attempt to create a perfect copy of the object they were trying to render.

**FIGURE 1:**
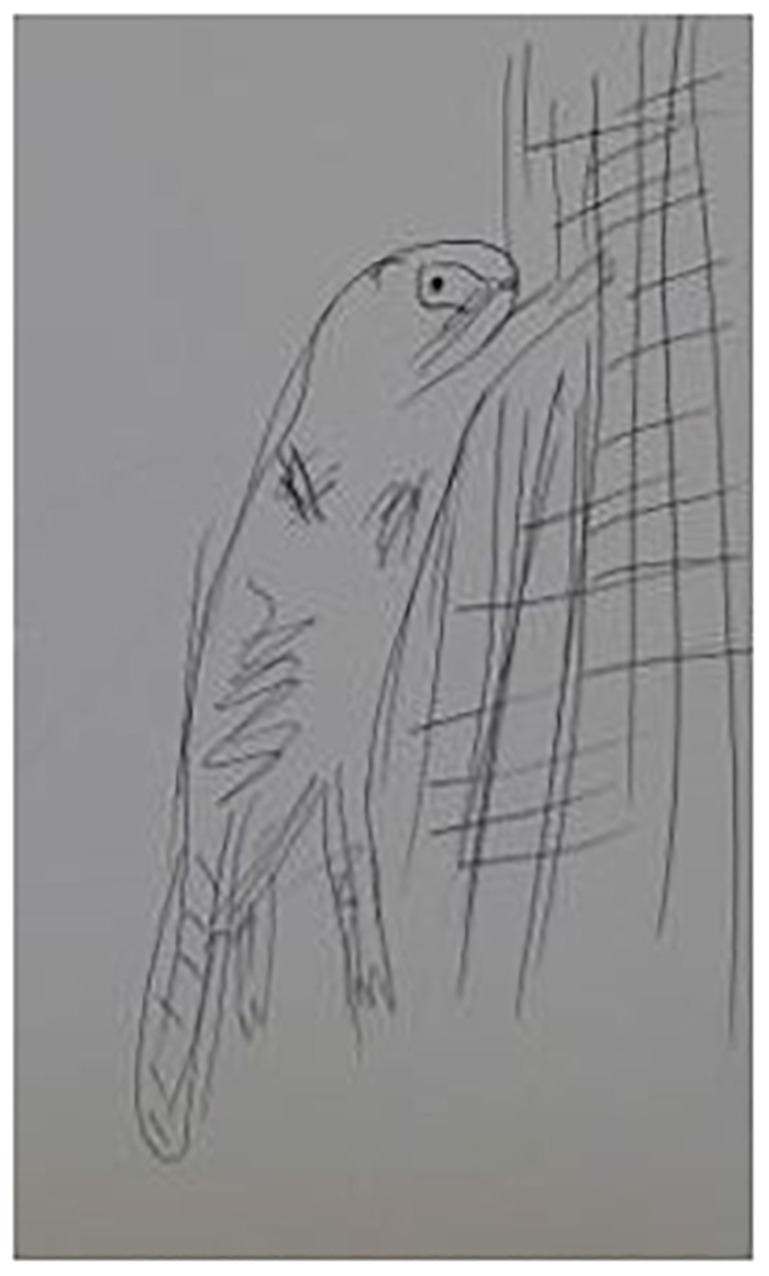
A ferret (participant 2).

**FIGURE 2:**
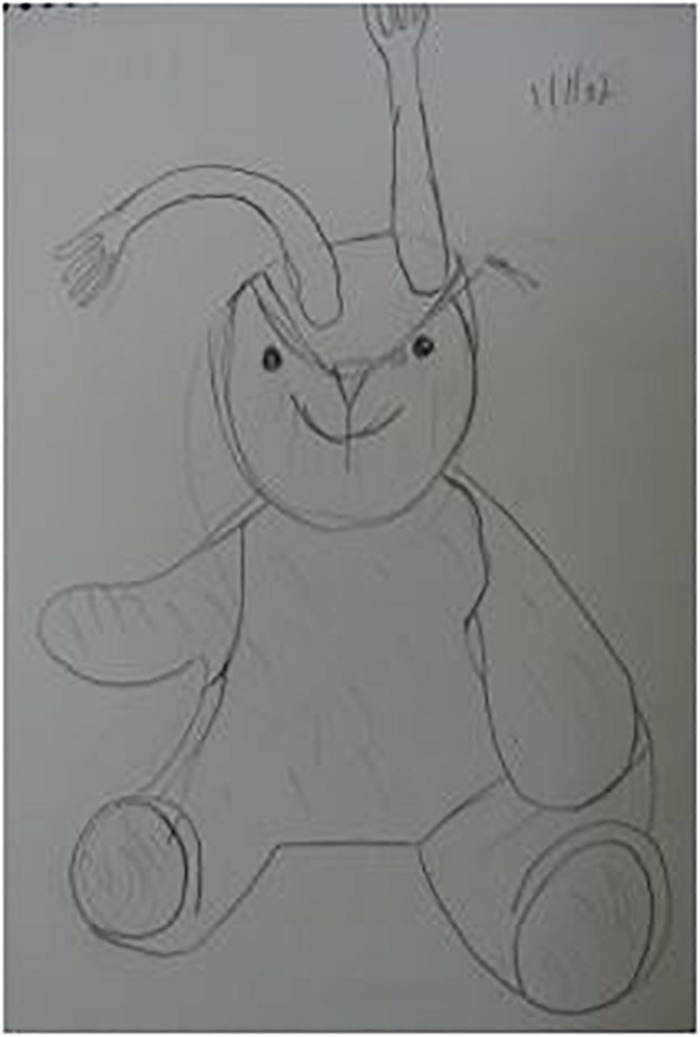
A rabbit with fork ears (participant 2).

Participant 2 (Figures 1, 2) discussed her scribbles and expressed the tension between harsh self-judgment and liberating playfulness:

… While I’m looking at most of the artworks, I see that they are not so serious, kind of a doodle … I know, though, that if I want to paint seriously, I can do so … On the other hand, it’s fun sometimes to scribble a little bit, a release, yes, you do not have to do anything specific, even if it does not come out well.

Participants highlighted an aesthetic approach that offered new insights into the question: What is beautiful? They moved from an objective approach that emphasized “serious” or “accurate” as being essential to their product, to a subjective approach that stressed “playful” and “liberating” as being essential. This shift also revealed a change in their ability to disconnect from an external “normalizing” gaze on their art, and revealed an inner, subjective gaze.

Finally, participants demonstrated how DFO could affect their consideration of their own art and how it had helped them move beyond their previous way of defining and appreciating aesthetics. Participant 1, for instance, when asked what she gained from the process of DFO, explained how she’d learned that what could have been considered “ugly” by her or by others, could, alternatively, have an aesthetic value. Choosing handwriting as an example, she said:

I once read that artists are supposed to have really beautiful handwriting. I have the ugliest handwriting in the world, but [through the DFO process] I realized that there are different kinds of beauty: There is the beauty of a flower and the beauty of a fire.

In sum, in the process of DFO, participants developed a flexible way of looking at aesthetics which may in turn have affected their self-perceptions. This shift may have led to a greater ability to accept their own imperfections.

### The Mindfulness Dimension: Between Merging and Separation

Participants often addressed different forms of what could be regarded as mindfulness and considered it an essential aspect of DFO. What we term “mindfulness” was described by the participants as a sense of increased concentration and a certain detachment from the external world, while merging themselves with the act of drawing. However, at the same time, and perhaps paradoxically, this mindfulness was also characterized by participants’ increased alertness to and awareness of their surroundings. Thus, we identified mindfulness in DFO as a process that involves movement between merging and separation. Essentially, this movement was associated with the actual act of observing/drawing, which demanded back-and-forth attention from an external image to an internal image. Participant 8, for example, described a feeling of getting inside herself and at the same time connecting to the environment:

I can observe for 4 h and draw one piece consecutively. I correct it, I concentrate, I think I’m actually closing myself off [to the outside]. I hear nothing, really. People could call my name, but I don’t hear a thing. …kind of being alone with myself. On the other hand, I sort of hear all the things that others are saying to me.

The merging aspect also included a feeling of “taking a break” from the external world. This feeling was described as a pleasant, relaxing feeling, and generally as something to aspire to. Several participants addressed this aspect:

Participant 3: It’s like having a break from all the other activities and having a new activity which is different. Like a screen-saver on a computer.…

Participant 7: [Observing the object] It kind of silences the thoughts. …it’s just like virtually giving an anesthetic to your thoughts.

Along with this break, the merging experience connected participants to themselves and made them aware of their most delicate bodily sensations. Participant 1 for instance described a sense of tension in her head when she was concentrating:

I felt at the time, when I was observing, as if I were entering into the lines of the picture … I could not just sit and draw. I had to concentrate very deeply, and I felt a sense of tension, which I felt in my head.

Participants explained that the act of observing an external object in DFO allowed their attention to be focused simultaneously on themselves and on the outside, thus enabling a unique space of mindfulness to come into being. The drawers noticed that on the one hand, the external, observed object marked a reality. On the other hand, they engaged in the process of DFO with a profound connectedness to their internal reality. An example can be seen in the words of Participant 6:

You paint something external, but you have something inside you, and your inner thing is reflected in your work.

In sum, DFO allowed flexible movement between merging with the object and separating from it, upon which participants could connect with their inner feelings and sensations.

### The Reflection Dimension: Between Similarities and Differences

The reflection dimension was related to participants’ search for themselves in their drawings or, at the least, for traces of their unique identity in their drawing style and product.

**FIGURE 3:**
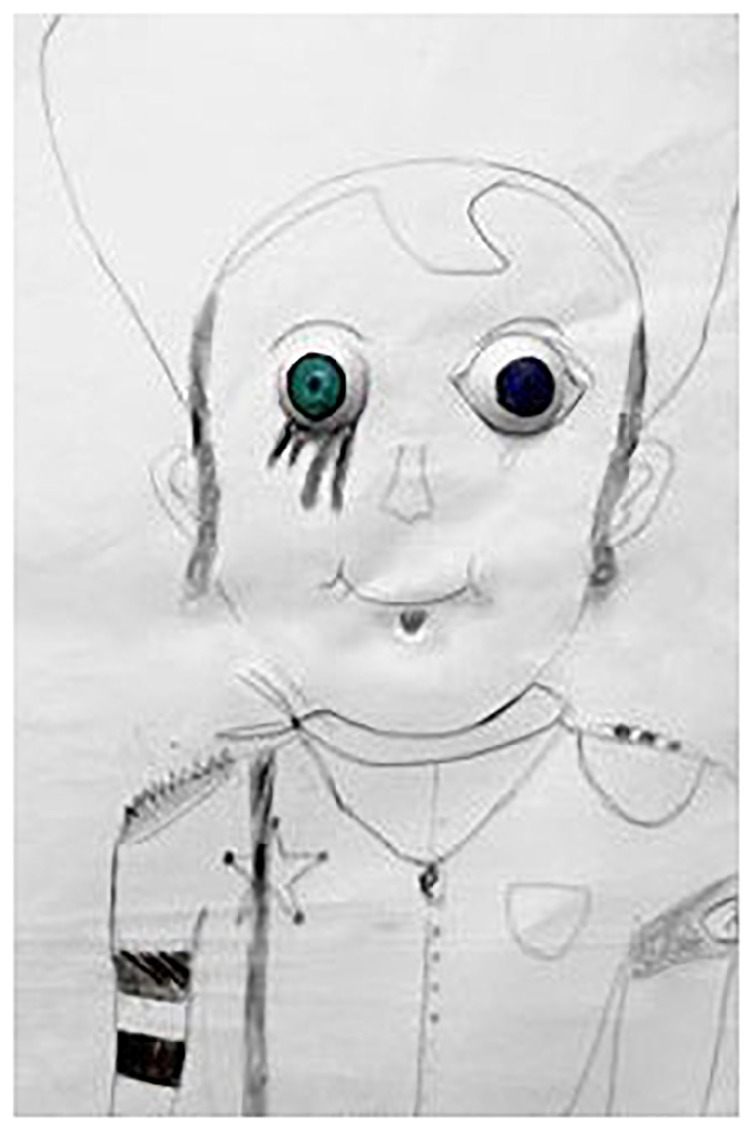
A cowboy figure (participant 4).

Participant 4, for instance, described how he recognized parts of his personality and history in a cowboy figure he drew (Figure 3):

[This drawn cowboy] has the face of a little baby, it has the wig of an Ultraorthodox Jew, it has a sheriff’s star, it has the spiky hairstyle of, what do they call it? A punk. I know now why it made me laugh. [It is] because when I was 16 years old, I often visited Jerusalem. Every 2 meters people would stop me and ask me if I was a punk, and I would tell them “no, no, no,” and there’s the cowboy’s hat. Who didn’t want to be a cowboy at least once when they were children? And the wig, it’s because I was religious… The swastika? I just discovered something about it: I discovered it after I left home, I discovered that my grandfather was in the Holocaust and I didn’t know that.

Identifying one’s personal narrative in one’s reflections about the drawing was facilitated through the drawing’s themes, as well as through the identification of one’s unique drawing style as a personal signature. Participant 8, for instance, learned to recognize her line-style, as well as to identify “foreign” lines that could not have been drawn by her.

It is clear to me that [lines that others draw] will be different because each one has his own unique line. … I recognize my line so well that I can tell where in the painting [the art therapist] drew and where I drew, because I see this line, because one can see the marking here of someone experienced and the marking of someone who just started to learn.

The recognition of the self was often mediated through the observed object, that served, perhaps, as a target for the projection of internal “materials.” In this regard, the process of drawing a neutral object enabled participants to consequently acknowledge and recognize the difference between “reality-object” and “drawing-self,” and thus to see how their own emotions and perceptions were projected onto the object. The reverse process also occurred, when drawers incorporated aspects of the object being drawn into their perception of self-identity. The object could, thus, become a source for generating new emotions and meanings.

Participant 9, for example, described how in the process of observing and drawing other participants’ faces, his own facial expressions and emotions ended up being reflected in the drawing. Similarly, he adopted and internalized his peers’ expressions and emotions. One notices, in his quote that follows, that it is difficult to distinguish between what belongs to the object – in this case, other participants’ faces – and what belongs to the self:

When I draw facial expressions, I often capture some kind of emotion without even noticing that I’ve captured it, for instance when I draw someone’s eyes, and there’s some kind of expression in his eyebrows … and then I notice that my eyebrows have the same expression, like this emotion passed to me, and if there’s a smile on the face of a person in a drawing, like, a particular smile, I begin to smile in the same way. It’s like I feel this expression subconsciously … it’s like what I feel is passed to the paper.

**FIGURE 4:**
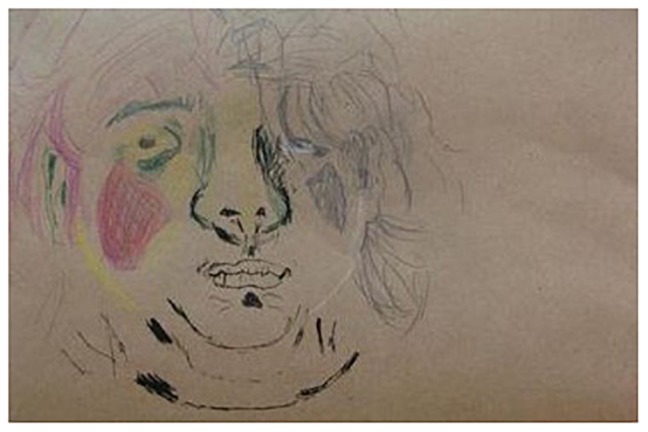
A self-portrait (participant 1).

Indeed, the drawn object was often perceived by participants as a mirror, but one that captured an internal reality more than an external one. This phenomenon was evident mainly in drawings that were defined as self-portraits, in which participants could learn about themselves by viewing the difference between the “object” (a photograph of themselves, which served as the object for drawing, for instance) and the actual portrait. For instance, Participant 1, in her self-portrait (Figure 4), expressed different parts of her personality, rather than her actual appearance:

Since it is a self-portrait, I didn’t just decide how to draw it. I also decided what facial expression [would be in it]. Well, I know that while I’m looking at it, I am seeing myself from the inside; I am aware of the fact that if I were to look in the mirror now, I would see something different.

Additionally, participants saw in the drawing materials elements of their personalities or of their mood, and they realized that some materials expressed their personalities and moods better than other materials. Participant 7 explains:

Although you gave me charcoal, and I didn’t touch it till now, it [the charcoal] is so me. [Back then, when] I took art lessons, I always chose watercolors. It doesn’t matter if I paint something in charcoal that is “open” and “sunny,” it will always come out as something that is not very naive. Watercolors are very polished and childish. Charcoal, like a piano, feels very old. charcoal will have something a little tired in it, maybe even depressing.

Insisting on a certain material, or on a certain drawing style, however, made participants feel at times that they were trapped in their tendency to draw what was familiar to them, or to use a style/material that “resembled” them in some way, thus emphasizing, perhaps, the challenge of getting to know and experience new, unfamiliar aspects of themselves.

In summary, the reflection dimension enabled participants to reflect upon identity and self, by using the object as a target of projection, and as a point of comparison.

## Discussion

Our results demonstrate that adolescents experienced three distinct tensions in the process of DFO: between self-acceptance and self-judgment, between merging and separation, and between similarities and differences. These tensions, we claim, might have a unique therapeutic value: they elicit a contemplation of issues related to identity and self, and may also promote the emotional well-being of participants through the processing of these tensions (see Table 3).

**TABLE 3:** A summary of the process and mental-health benefits of DFO as described by adolescents.

DFO dimension	Main focus	Mental health benefits (emotional well-being)
*Aesthetic* between self-acceptance and self-judgment	Accepting and acknowledging different kinds of aesthetics as valid.	Reduction of self-criticism. Developing a positive body image.
*Mindfulness* between merging and separation	Being mindful of the self as well as of the object in the process of drawing.	Connecting to one’s inner feelings and sensations, while not over-identifying with them or being absorbed by them.
*Reflection* between similarities and differences	Reflecting on self with regards to the process (e.g., materials) and product of drawing (e.g., significance and meaning of drawing).	Getting to know and experience new, unfamiliar aspects of self. Enhancing self-acceptance.

Adolescents’ perspectives on seeing, contemplating, and maybe judging different selves in the representation of the object echo psychological theories on adolescence as a period during which self-exploration, self-discovery, and identity formation take place (Blos, 1967; Kroger, 2005). Our results, however, further suggest that DFO might be considered a containing and distancing mechanism for this exploration through the object/subject dichotomy and as a safe projection of personal issues onto an artistic process. We consider this process to be safe, as the object is both external and internal (Harty, 2012); clients, therefore, have some agency in deciding the degree to which they identify with it and the degree to which they reject it.

Although one might claim that every CAT offers this kind of mechanism (see, for instance, Potash, 2009), we suggest that the existence of an identifiable external object in DFO makes the method more amenable to enabling this distancing of self from the product, while also enabling the individual to recognize the represented self in it. In the highly emotional period of adolescence (Cummings et al., 2014), this mechanism can be truly valuable.

Indeed, Ramm’s (2005) description of DFO as “inner thought” aligns with our findings on the drawn object as reflecting an inner reality but not imposing it on the individual. It seems that although the object exists “on its own,” it also functions as a mirror and is thus a target for projection. These ideas also correspond with Fougner and Kordahl’s (2012) findings that observational drawing allows one to relate to the observed object both as an object and as a subject, thus facilitating self-reflection.

Our findings point to an interesting phenomenon in which some participants tend to both project themselves onto the object, as well as to internalize the object and change themselves through it. For example, they adopt another person’s (object of drawing) mood and/or facial expression. Thus, Wix’s (2009) notion of an empathic aesthetic could be expanded to include not only projection but also introjection. Therapists could make use of the projection and introjection processes that take place with an object, in order to allow clients flexibility and attentiveness to different possibilities of being.

The processes of projection and introjection that take place with an object also involve an aesthetic judgment and comparison with drawings made by peers in the group. Indeed, it is well-established that the peer group has a crucial influence on adolescents’ identity and behavior (Dumas et al., 2012), sometimes in a negative sense. Thus, the aesthetic dimension, while serving as a basis for judgment and competition, could also provide leverage for processing issues related to the development of self-compassion and self-acceptance (see, Neff and McGehee, 2010). When engaging in DFO, self-compassion and acceptance could be accompanied by a loving, flexible gaze directed toward both the aesthetics of the final product, as well as toward the creative self and the creative process (see also, Marsh et al., 2017). Moreover, focusing on the aesthetics of the visual object might allow for the adoption of a flexible perception toward the concept of beauty, which could become a way to process and contain the extensive preoccupation that adolescents have with their physical selves (Hargreaves and Tiggemann, 2004; Burnette et al., 2017).

Non-judgmental identity contemplation cannot be understood, however, without taking into consideration the idea that such contemplation is based on a mindful liminal experience of both being and not being “me.” In this regard, our results align with Milner’s (2005a,b) psychoanalytic conceptualization of CAT, mainly with her idea of drawing as opening a mindful space for playfulness and creativity (see also Cain, 2006). Mindfulness, as such, is promoted through the act of drawing and affects participants’ well-being outside the group work. It is important that therapists be aware of this mechanism and encourage participants to explore it in their lives beyond the DFO sessions.

The observational mechanism of being both the object and the subject of drawing invited drawers to become “external” and perhaps retrospective witnesses of themselves (Yaniv, 2012). The idea of witnessing has been previously explored in the CAT literature regarding the therapist-client dyad; seeing participants in CAT groups as witnesses of others in their group (Peleg et al., 2014; Keisari et al., 2018), and as external witnesses of artworks created in the context of CAT (Knill, 2011; Barak and Stebbins, 2017). However, DFO expands this notion to include not only the witnessing of self and others, but also the witnessing of an actual object. Our results suggest that through the processing of a benevolent witnessing gaze toward the object of drawing, and toward the drawing as object, participants can process their perspectives on self, potentially toward the development of a positive self-image.

### Practical Implications

Practical implications can be drawn from the three dimensions highlighted in this article, namely: the aesthetic, mindfulness, and reflection. Based on the aesthetic dimension, art therapists should be aware of instances in which clients are critical of their drawing. Art therapists could suggest to their clients that they observe the object in different ways, focusing, for instance, on texture, zooming in, or zooming out. Alternatively, art therapists could encourage clients to use scribbling, rather than attempting to copy the object, while instructing them to observe an inner object – that is, an object that they imagine based on the object at hand. Such exercises could help clients adopt a more flexible gaze on what is a “beautiful” or a “good” drawing, and by doing so, potentially allow for a less critical viewpoint on other aspects of their lives.

In order to promote mindfulness, art therapists could encourage their patients to explore the here and now of the act of drawing. Such mindfulness could be encouraged by instructing clients to focus their attention not on their expectations of a future product, or on negative accruing thoughts and feelings, but rather on the present moment of the act of drawing. Based on such experiences, therapists could engage clients in a discussion about mindfulness, as a kind of attentive, non-judgmental awareness of the present moment. Practicing mindfulness in DFO sessions might very well become a resource for clients to use in other aspects of their lives.

As for reflection, therapists can offer their clients a chance to think about how they perceive their drawings as a manifestation of their identity, and within this search, to see if they can identify their unique artistic “fingerprint.” As this fingerprint is likely composed of different materials, line styles, colors, and composition, searching for these identifiers within the artistic product could encourage reflection on issues of identity. Art therapists, encouraging discussion, further exploration of, and experimentation with new modes of artistic expression, might enable an “opening up” of new reflections on self-identity.

## Limitations and Conclusion

This research study has a few limitations: (1) The researcher who conducted the interviews was also the art therapist who conducted the DFO groups (see section “Methodology” and “Ethical Consideration”). This factor might have influenced participants’ ability to be completely open in their expressions. However, the prior acquaintance and already-established trust may have allowed for a confident sharing of personal views. Moreover, cooperation between the three researchers during the analysis reduced potential bias. (2) Although we referred to some possible influences that being in a group may have had on the study’s results, it is possible that the group influence had an even greater effect than was assumed here. While this is not necessarily a limitation, but rather pertains to the scope of this particular paper, it remains for future studies to further explore DFO in individual settings as well. (3) Our study is based on a small sample of adolescents from Israel and, as such, a different sample from a different country might have yielded different conclusions. Research studies from other parts of the world could broaden the picture we currently have on this topic with perspectives from different contexts and cultures. However, although the generalizability of this study might be limited, given that research on this subject is scarce, it can nevertheless provide important knowledge that could inform other researchers.

In sum, this research provides a road map of adolescents’ perspectives on DFO, a subject that has not yet been explored in the CAT literature. As DFO could make a profound contribution to adolescents’ mental health and their emotional well-being, we call upon other researchers to provide their own accounts of DFO in therapeutic settings and expand our knowledge on DFO and emotional well-being.

## Data Availability Statement

The datasets generated for this study will not be made publicly available, as they contain information that could compromise the privacy of research participants. Requests to access the datasets should be directed to the corresponding author.

## Ethics Statement

The studies involving human participants were reviewed and approved by the Ethics Committee of the University of Haifa, Israel. Participants provided written consent to participate in the research. For minors, parental consent was obtained.

## Author COntributions

All authors conceptualized and coordinated the study, participated in the study design, data analysis, data interpretation, and writing. NW collected the data. AB and DY revised the preliminary drafts and supervised the research process.

## Conflict of Interest

The authors declare that the research was conducted in the absence of any commercial or financial relationships that could be construed as a potential conflict of interest.
